# Gallbladder volvulus and hernia through the foramen of Winslow: a case report

**DOI:** 10.1093/jscr/rjaa424

**Published:** 2020-10-31

**Authors:** Toan Huy Nguyen, Kinh Huy Tran, Xuan Anh Le, Huong Van Nguyen, Quyet Van Ha

**Affiliations:** 1 Department of General Surgery, Nghe An General Hospital, Nghe An Province, Vietnam; 2 Department of General Surgery, Ha Noi Medical University, Ha Noi City, Vietnam

## Abstract

Gallbladder hernia through the foramen of Winslow is an uncommon condition and gallbladder hernia combined with volvulus is even rarer. A 70-year-old patient was hospitalized with the clinical signs of pain in the right hypochondriac region associated with fever. The computed tomography scan images showed some signs of gallbladder herniation through the foramen of Winslow. We decided to remove the gallbladder and found the gallbladder infundibulum twisted and necrotic. This was the first case of a male patient who suffered from gallbladder herniation with volvulus after three cases of female patients reported in the literature.

## INTRODUCTION

Internal hernia through the foramen of Winslow (epiploic foramen) was first recognized and reported in 1834 by Bladin. This is a very rare type of internal hernia, which only accounts for 8% of all internal hernias [1]. Gallbladder volvulus is also an uncommon disease, which was first described by Wendel in 1989 and there were only 500 reported cases in the English literature [2, 3]. Only 3 previous cases of internal herniation of the gallbladder through the foramen of Winslow with gallbladder volvulus have been reported in the literature [4–6]. Recently, although imaging modalities have been significantly improved, making an accurate diagnosis of these cases before surgery is still a challenge for clinicians.

## CASE REPORT

A 70-year-old male patient, with a past medical history of chronic obstructive pulmonary disease and no previous abdominal surgery, was hospitalized with the chief complaint of pain in the right hypochondriac region, fever (38°C). The clinical examination showed that the patient was conscious and had slight jaundice. The gallbladder could not be palpated in the right hypochondriac region and abdominal guarding was present.

The blood count results were as follows: red blood cell: 4.89 T/L; white blood cell: 22.32 G/L (Neutrophil: 91%); platelet: 329G/L; hematocrit: 28.5%; prothrombin (PT): 93.4%. The biochemical analysis showed: creatinin: 70 μmol/l; albumin: 38 g/l; total bilirubin: 18.9 μmol/l; serum glutamic-oxaloacetic transaminase (SGOT): 14 U/L (< 35); serum glutamic-pyruvic transaminase (SGPT): 42 U/L (<35); carcinoma embryonic antigen (CEA) 2.5 ng/ml; Ca-199 38.5 U/ml.

**Figure 1:**
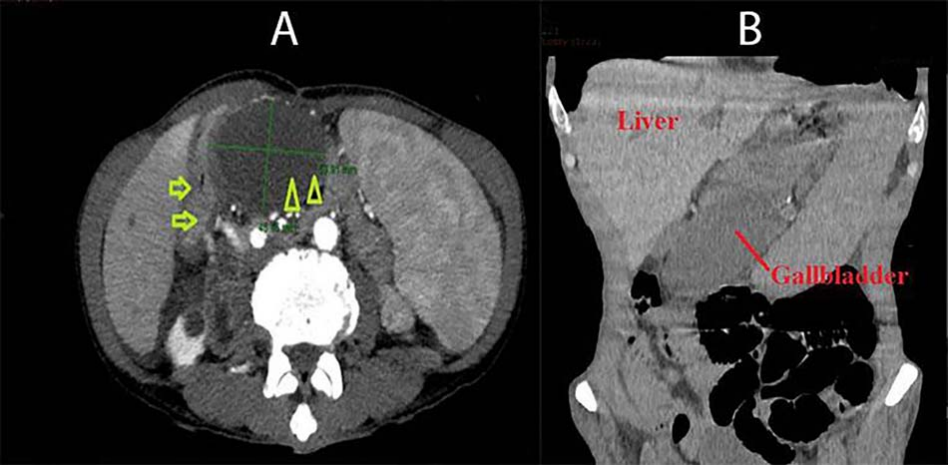
Abdominal CT images: **A** (axial view): duodenum (arrow) and the gallbladder located on the left of the duodenum and posterior to the stomach (arrow head); **B** (coronal view): the gallbladder was situated under the stomach and far from the gallbladder bed.

The abdominal computed tomography (CT) showed that the gallbladder was enlarged and distended, with 55 mm in transverse diameter and 7 mm in wall thickness. The gallbladder was seen inside the lesser sac and on the left of the D2 part of the duodenum. It also pushed the stomach anteriorly and superiorly (Fig. 1). The diagnosis of cholecystitis and gallbladder hernia through the foramen of Winslow was made.

**Figure 2:**
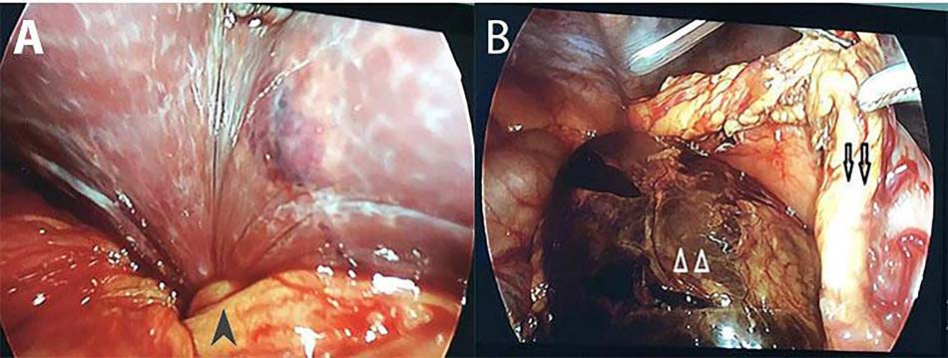
Intra-operative photographs: A: the torsion point at the gallbladder infundibulum (black arrow head); B: necrotic gall bladder (white arrow heads) located posterior to the stomach (black arrows).

An emergency operation was indicated. Initially, we approached the abdominal cavity with laparoscopy and the gallbladder was not seen in its normal location. Then, the greater omentum was opened and the stomach was retracted superiorly. The gallbladder was found to herniate into the lesser sac through the foramen of Winslow. The infundibulum was twisted and the whole gallbladder was necrotic with darkened color (Fig. 2). We attempted to bring the gallbladder back to the normal position through the foramen of Winslow with laparoscopy but we failed to do so. Therefore, we decided to shift to laparotomy and saw the gallbladder, which was rotated three times counterclockwise, through the foramen of Winslow. The width of the foramen was ~4 cm. Then, the gallbladder was removed and the foramen of Winslow was narrowed with the greater omentum. A suction drain was placed in the hypochondriac region. The patient had an uneventful postoperative course and was discharged on postoperative Day 10. The histopathology was cholecystitis.

## DISCUSSION

Regarding gallbladder hernia into the lesser sac via the foramen of Winslow, since the first case reported by McCrae in 1951, there were only 13 reports of operated cases and all of them were females. Among these patients, only three cases were associated with gallbladder volvulus and all of them were cholecystitis which was confirmed by post-operative histopathology [7]. Our patient was the fourth reported case of gallbladder volvulus combined with herniation through the foramen of Winslow.

Because of the difficulty in making a diagnosis of internal hernia, the role of imaging modalities is very important. In the past, these herniations were often assessed with conventional abdominal X-ray with contrast. Recently, CT scan has become the first choice of imaging for these patients thanks to its feasibility, short processing time and its ability to reconstruct the abdominal organs [8]. The typical findings of gallbladder hernia through the foramen of Winslow in CT scan are as follows: the presence of the gallbladder in the lesser sac, between the inferior vena cava and the hepatoduodenal ligament; the image fluid-filled cyst in the lesser sac with an end near the anterior aspect of the foramen of Winslow [7].

In our particular patient’s case, the clinical findings did not show anything special. There was only continuous pain in the right hypochondriac, in combination with sepsis syndrome. On imaging, the radiologists suggested the image of gallbladder hernia through the foramen of Winslow. Nevertheless, the diagnosis of gallbladder volvulus could only be made intra-operatively. Not all cases of torsion can be identified in pre-operative CT scan images, even when there is no herniation. It was suggested that it would be more feasible to diagnose gallbladder volvulus if CT-guided puncture with contrast injection was used [6] because the contrast agent could not enter the main bile duct or the intra-hepatic bile duct and would be stuck at the infundibulum. However, in case when the patient comes to see clinicians with acute abdominal pain and requires surgically intervened as soon as possible, the use of these sophisticated diagnostic measures is not practical. Furthermore, the data also showed that only 1% of all volvulus cases was diagnosed pre-operatively [9].

## CONCLUSIONS

CT scan is the first tool of choice in diagnosing gallbladder hernia. However, a clear view of the gallbladder infundibulum is not always achieved. Preoperative CT-guided puncture with contrast injection of the biliary tract could be a helpful method to accurately determine the torsion of the gallbladder infundibulum.

## CONFLICT OF INTEREST STATEMENT

None declared.

## Supplementary Material

Part_1_laparoscopy_rjaa424

Part_2_Laparotomy_rjaa424
